# Metabolic reprogramming in melanoma therapy

**DOI:** 10.1038/s41420-025-02617-3

**Published:** 2025-07-05

**Authors:** Dongliang Shen, Lu Zhang, Shun Li, Liling Tang

**Affiliations:** 1 Key Laboratory of Biorheological Science and Technology, Ministry of Education, College of Bioengineering, Chongqing University, Chongqing, China; 2 Department of Immunology, School of Basic Medical Sciences, Chengdu Medical College, Chengdu, Sichuan China

**Keywords:** Cancer therapy, Cancer metabolism, Melanoma

## Abstract

Melanoma, a deadly and aggressive cancer, exhibits significant metabolic reprogramming that supports energy production, biosynthesis, and tumor progression. This metabolic adaptation drives melanoma growth, proliferation, metastasis, and therapy resistance, highlighting its potential as a promising target for therapeutic intervention. This review focuses on the latest studies elucidating metabolic pathways involved in melanoma progression, therapeutic response, and resistance. Additionally, the potential of targeting metabolic pathways–either alone or in combination with established therapeutic inhibitors–to block disease progression in melanoma is also discussed. Such insights might improve our understanding of metabolic pathways in melanoma development and foster advancements in melanoma therapy.

## Facts

- Melanoma demonstrates metabolic plasticity, reprogramming its metabolism in response to tumor stage and environmental challenges.
- Melanoma cells at metastatic and therapy-resistant stages display increased oxidative phosphorylation and a heightened capacity to maintain redox balance.
- Targeting metabolic pathways offers a promising strategy to improve the effectiveness of melanoma therapy.

## Introduction

The frightening reason for tumors lies in their highly proliferative and metastatic potential, which is, to a large extent, driven by their distinct metabolic pathways. Under normal conditions with enough oxygen, cells preferentially utilize oxidative phosphorylation (OXPHOS) to acquire energy due to its high efficiency in generating adenosine triphosphate (ATP), the molecular form of stored energy, compared to other metabolic pathways. Glycolysis is an alternative pathway for obtaining ATP in response to the rapid consumption of oxygen. Although glycolysis provides extremely less energy than OXPHOS, it allows cells to rapidly adapt to anaerobic environments rather than undergoing cell death [1]. However, the metabolic pathways in cancer cells are different. Tumor cells require a larger quantity of energy to meet the demands of rapid growth. Theoretically, the first choice of metabolic pathways for tumor cells should be OXPHOS. Surprisingly, they prefer glycolysis even under aerobic conditions, a phenomenon known as aerobic glycolysis or the Warburg effect. When this phenomenon was first discovered in the early 1920s, the hypothesis explaining this metabolic reprogramming from OXPHOS to glycolysis was mitochondrial dysfunction [2]. However, an enormous number of studies have overturned this hypothesis by demonstrating the existence of functional mitochondria in tumor cells. With the advancement of metabolic mechanisms, researchers have gradually realized that, rather than solely acquiring energy, tumor cells primarily generate a diverse array of metabolites to support biosynthesis and cellular functions, such as proliferation and metastasis. In cancer biology, this kind of metabolic reprogramming is a hallmark of tumorigenesis and progressive disease. Cancer cells dynamically alter their metabolic pathways to meet the energy and biosynthetic demands required for growth, survival, and adaptation at different stages of tumor development [3–5]. Moreover, a significant increase in studies has demonstrated that targeting metabolism remarkably influences tumor progression and treatment response across various cancer types [6–9].

Melanoma is a highly aggressive cancer with formidable lethality due to its phenotypic plasticity. Glycolysis serves as the central metabolic pathway in melanoma, driving ATP production and the synthesis of nucleotides, amino acids, and lipids essential for cell survival, growth, and proliferation [10–12]. However, melanoma metabolism is highly dynamic and heterogeneous. Metastatic melanomas and targeted therapy-resistant melanomas exhibit dramatically increased OXPHOS in experimental models and clinical samples [13–16]. The ratio of glycolysis to OXPHOS in advanced melanoma is higher than in early metastatic melanoma [17]. Targeting the dynamic metabolism is a promising strategy to control melanoma progressive disease and improve response to therapy. Here, we review the metabolic reprogramming in melanoma tumorigenesis, dissemination, progression, and drug resistance. Additionally, potential therapeutic approaches targeting metabolic shifts for melanoma therapy are also discussed.

## Metabolism in melanoma tumorigenesis and growth

### Glycolytic reprogramming in melanoma

The dominating metabolic pathway in melanoma is glycolysis, a hallmark of its metabolic reprogramming. Several key genes, including some melanoma driver mutation genes, play a major role in regulating this metabolic shift and supporting tumor progression. Approximately half of melanomas harbor the v-Raf murine sarcoma viral oncogene homolog B (BRAF) mutation, a pivotal driver gene in the mitogen-activated protein kinase (MAPK) signaling pathway. BRAF^V600E^ mutation, which shows activated kinase activity, enhances glycolysis and suppresses oxidative phosphorylation (OXPHOS), promoting metabolic reprogramming that supports tumor growth and survival. P90 ribosomal S6 kinase (RSK), a major MAPK substrate, is necessary to maintain glycolysis metabolism in BRAF-mutated melanoma [18]. RSK directly phosphorylated 6-phosphofructo-2-kinase/fructose-2,6-bisphosphatase 2 (PFKFB2), an enzyme that catalyzes the synthesis of fructose-2,6-bisphosphate during glycolysis. The enzyme-dead mutation of PFKFB2 suppresses glycolysis, leading to the significant reduction of tumor growth in nude mice, while not impairing the proliferation of melanoma cells under normal in vitro culture conditions. Concurrently, BRAF suppresses oxidative metabolism through distinct mechanisms. It downregulates the expression of peroxisome proliferator-activated receptor gamma coactivator 1-alpha (PGC1α), a master transcriptional regulator of mitochondrial genesis, through the microphthalmia-associated transcription factor (MITF) [19]. Therefore, blocking BRAF kinase function with BRAF inhibitors reduces glycolysis and induces oxidative phosphorylation, which is promising in preventing melanoma progression.

**Fig. 1:**
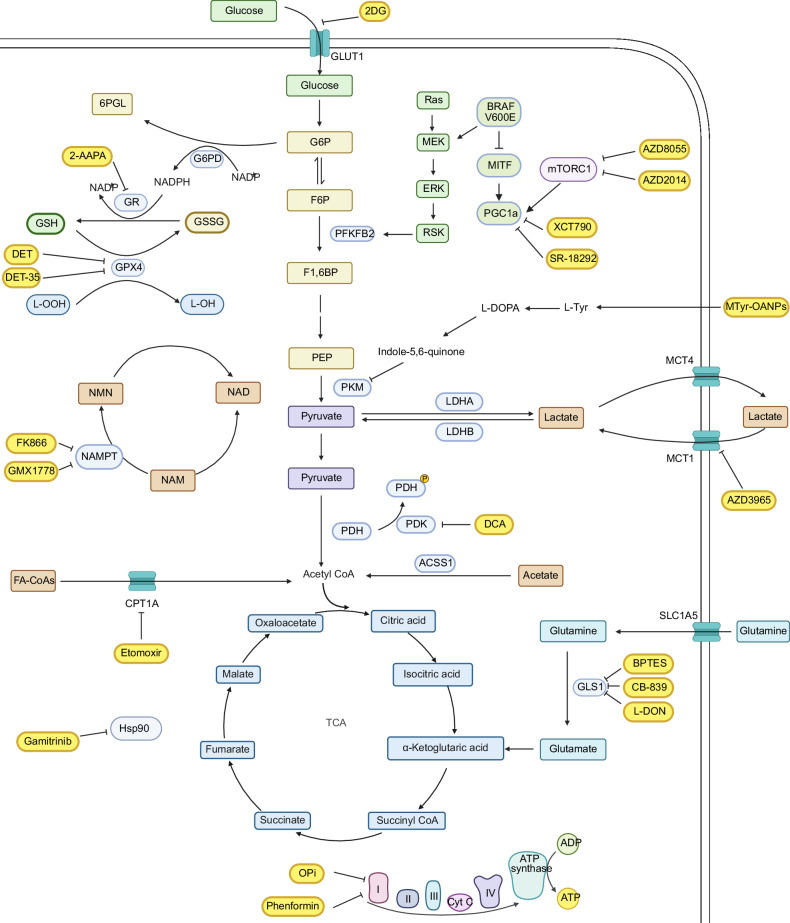
Inhibitors of metabolism in melanoma therapy. This schematic illustrates key metabolic pathways in melanoma, highlighting potential therapeutic targets and inhibitors. The glycolytic pathway, TCA cycle, glutamine metabolism, and oxidative phosphorylation are shown with their respective enzymes and metabolites. Specific inhibitors targeting critical metabolic nodes are marked in yellow. These include 2DG (glucose metabolism), AZD3965 (MCT1), BPTES/CB-839/L-DON (glutaminase), Etomoxir (CPT1A), and OPi/ Phenformin (oxidative phosphorylation). Additionally, inhibitors affecting the redox balance (2-AAPA, DET, DET-35), NAD metabolism (FK866, GMX1778), PGC1α (XCT790, SR-18292), PDK (DCA), and mTOR signaling (AZD8055, AZD2014) are depicted.

Furthermore, targeting glycolysis, a metabolic pathway heavily relied upon by melanoma for survival and proliferation, represents an encouraging therapeutic strategy to slow down melanoma growth and enhance antitumor efficacy. 2-Deoxy-D-glucose (2DG), a well-known glucose analog as a potential glycolysis inhibitor, interrupts glucose metabolism by targeting glucose uptake, effectively decreasing melanoma growth [20] (Fig. 1). Nowadays, an increasing number of studies have illustrated the crucial role of 2DG in the initiation and progression of melanoma. For instance, when combined with coronatine, a regulator of secondary metabolite biosynthesis, 2DG demonstrates enhanced efficacy in reducing the proliferation of metastatic melanoma cells [21]. Additionally, 2DG also increases oxidative stress and induces autophagy [20], further contributing to its antitumor effects. Moreover, in combination with cisplatin (a chemotherapy drug) or staurosporine (a protein kinase inhibitor), 2DG dramatically induced apoptosis by disrupting mitochondrial homeostasis in human metastatic melanoma cell lines [11]. Similarly, combining 2DG with pyruvate (Pyr) promotes apoptosis through increasing oxidative stress. Additionally, it also enhances tumor necrosis factor-related apoptosis-inducing ligand(TRAIL)-induced apoptosis through X-box binding protein 1 (XBP-1)-mediated upregulation of TRAIL receptor 2 [22]. Surprisingly, the response period and antitumor efficacy are incredibly improved by delivering 2DG and doxorubicin (DOX) together in a cellulose nanocrystal (CNC)-annealed hydrogel (CAH) structure [23]. Metabolism reprogramming induced by 2DG makes the neuroblastoma RAS viral oncogene homolog (NRAS) mutant melanoma cells sensitive to BRAF inhibitor (BRAFi) sorafenib [24]. Besides 2DG, the supplementation of L-Tyrosine, another candidate for inhibiting glycolysis, strongly impedes melanoma proliferation by reactivating melanogenesis-related metabolism through L-Tyrosine-oleylamine nanomicelles (MTyr-OANPs) [12] (Fig. 1). The combination of MTyr-OANPs and photothermal therapy completely eradicated tumors in B16F10 tumor-bearing mice.

### Non-glucose carbon sources fueling the TCA cycle

However, metabolism is not strictly glycolytic, as the tricarboxylic acid (TCA) cycle is functional even under hypoxia [25]. When glucose is primarily converted to lactate, glutamine serves as one alternative carbon source, fueling the TCA cycle [25–27]. This glutamine-driven TCA cycle activity supports critical biosynthetic pathways, including fatty acid synthesis and the production of non-essential amino acids, to sustain tumor growth and proliferation. Glutamine metabolism not only supports the tricarboxylic acid (TCA) cycle but also reduces glutathione (GSH) synthesis and increases lipid hydroperoxides [28, 29]. These changes promote lipid peroxidation, a vital process involved in ferroptosis, an iron-dependent form of cell death. It has been demonstrated that enhancing glutamine metabolism by increasing solute carrier family 1 member 5 (SLC1A5), a glutamine transporter, boosts the antitumor activity of ferroptosis-induced compounds erastin and RAS-selective lethal compound 3 (RSL3) both in vitro and in vivo [28]. Additionally, dietary glutamine supplementation inhibits tumor growth in a transgenic melanoma mouse model [29].

Not only glutamine, but acetate also serves as an alternative carbon source, fueling the TCA cycle. This process is driven by acetyl-coenzyme A (CoA) synthetase 1 (ACSS1), which is highly expressed in melanoma [30]. ACSS1 facilitates the utilization of acetate in the TCA cycle and rewires glucose metabolism, contributing to the alteration of the cellular redox state. ACSS1 knockdown inhibits both primary and metastatic melanoma tumor growth in NSG mice. All the above studies underscore the importance of metabolic rewiring in melanoma initiation and local lesion growth. These findings highly encourage further exploration of its role in distant metastasis.

## Metabolism in melanoma metastasis

### OXPHOS activation and metastatic potential

Numerous studies have demonstrated distinct metabolic differences between primary and metastatic melanoma. For example, metastatic melanoma cells, such as 4C11+, exhibit enhanced oxidative metabolism, which is primarily driven by glutamine oxidation rather than pyruvate catabolism [13]. Additionally, stem-like cell subsets of A375 and WM115 display increased mitochondrial biogenesis and oxidative metabolism, which are dependent on PGC1α for maintaining stem-like features [31]. Targeting PGC1α with XCT790 or SR-18292 decreased mitochondrial mass and activity, effectively impairing clonogenic ability and invasion of melanoma cells [31] (Fig. 1). Salhi et al. further supported these findings by showing the upregulation of OXPHOS in invasive melanoma using a zebrafish tumor model [14]. Their study revealed that melanoma cells utilize PGC1α to enhance OXPHOS and ATP production, thereby promoting an invasive phenotype. In patient-derived xenograft (PDX) melanoma models, metastatic melanoma shows reduced expression of glycolytic genes and elevated expression of TCA cycle genes [15]. This metabolic shift is linked to the downregulation of the short isoform of glyceraldehyde-3-phosphate dehydrogenase, spermatogenic (GAPDHS). Reduced GAPDHS activity leads to decreased pyruvate carboxylase function and impaired aspartate synthesis, ultimately altering the metabolic profile of metastatic melanoma. The OXPHOS activity in metastatic melanoma relies on glutamine oxidation rather than pyruvate oxidation. Bis-2-(5-phenylacetamido-1,3,4-thiadiazol-2-yl)ethyl sulfide (BPTES), a selective inhibitor of glutaminase 1 (GLS1), dramatically inhibited the migration of metastatic melanoma cells in vitro [13] (Fig. 1).

### Antioxidant capacity and survival

High OXPHOS activity is often associated with elevated production of reactive oxygen species (ROS). To counteract oxidative stress and maintain cellular homeostasis, cells rely on protective mechanisms such as the pentose phosphate pathway (PPP), which generates nicotinamide adenine dinucleotide phosphate (NADPH) to regenerate GSH and combat oxidative damage. The capacity to withstand oxidative stress is critical for the metastatic potential of melanoma cells [32–35]. Redox imbalance caused by microsomal glutathione S-transferase 1 (MGST1) knockout effectively impaired melanoma metastasis [36]. Loss of glucose-6-phosphate dehydrogenase (G6PD), a key enzyme in the pentose phosphate pathway, reduced melanoma metastasis without obvious impact on the formation of primary subcutaneous tumors [34]. Similarly, inhibiting glutathione reductase (GR), which converts oxidized glutathione (GSSG) to GSH, with 2-AAPA induced oxidative stress, leading to the suppression of melanoma metastasis in vitro and in vivo [33]. However, treating melanoma cells with the antioxidant, N-acetylcysteine (NAC), diminished the efficacy of 2-AAPA in B16F10 cells [33] and promoted distant metastasis in NSG mice [32].

Fatty Acid Oxidation (FAO) has been shown to be an important source of NADPH. In clinical melanoma samples, metastatic melanoma exhibited elevated FAO and tectonic family member 1 (TCTN1) [37]. Mechanistically, TCTN1 enhanced FAO activity by binding to hydratase subunit A (HADHA) and subunit B (HADHB), key enzymes in the FAO pathway, thereby supporting circulating melanoma cell survival [38]. Noteworthy, FAO also promoted melanoma cell migration by inhibiting the formation of autophagosomes, independently of its roles in energy production or redox homeostasis [39]. Moreover, folate metabolism also provided critical intermediates for NADPH production and GSH synthesis in metastatic melanoma cells [32]. Targeting the folate pathway by knocking down related genes, such as aldehyde dehydrogenase 1 family member L2 (ALDH1L2) or methylenetetrahydrofolate dehydrogenase 1 (MTHFD1), or using methotrexate significantly inhibited distant metastasis [32].

Additionally, redox homeostasis is also regulated by selenocysteine-containing proteins [40], such as selenoprotein O, which promotes cancer metastasis by modulating mitochondrial function and oxidative stress [41]. The translation of selenocysteine-containing proteins requires Um34, a unique modification of the anticodon loop on tRNA^Sec^, which is controlled by FTSJ1, an Um34 methyltransferase highly expressed in metastatic melanoma. Furthermore, mitochondrial Ca^2+^ is an important regulator of mitochondrial metabolic output and redox signaling. The mitochondrial calcium uniporter (MCU) complex serves as the primary route for calcium entry into the mitochondrial matrix. High expression of MCU_A_ increased mitochondrial Ca^2+^ and oxidative stress, resulting in the reduction of the aggressive behavior of melanoma cells [42]. The ability to mitigate oxidative stress, which allows the increase of OXPHOS activity, is critical for the survival and metastatic potential of melanoma.

### Metabolic plasticity in advanced melanoma

Although metastatic melanoma exhibits high OXPHOS activity, glycolysis is also involved in melanoma progression and metastatic processes. In advanced metastatic melanoma, glycolysis is markedly upregulated under hypoxic conditions, particularly in the tumor core. This metabolic shift is regulated by lactate dehydrogenase (LDH) isoenzymes and monocarboxylate transporters (MCTs), such as MCT1 and MCT4 [17]. These components mainly participated in the regulation of glycolytic flux, lactate production, and pH levels to drive tumor progression and metastasis. Furthermore, the switch from OXPHOS to glycolysis has also been observed in tumor-repopulating cells (TRCs), a stem cell-like subpopulation with highly invasive phenotypes that are responsible for tumor regeneration, by regulating phosphoenolpyruvate carboxykinase 1 (PCK1) and PCK2 [43, 44]. Similarly, S100A4 increased glycolysis by blocking mitochondrial respiration, supporting the emergence of an invasive phenotype in melanoma cells [45]. Metabolic plasticity in advanced melanoma enabled tumor cells to adapt to diverse microenvironments, support metastatic dissemination and proliferation, and develop resistance to therapy [46, 47].

## Metabolism and targeted therapy

### Metabolic targeting to enhance therapeutic response

Melanoma is the most aggressive form of skin cancer, with more than half of cases harboring BRAF mutations [48]. The mutant BRAF exhibits constitutive activation of its kinase activity, leading to hyperactivation of MAPK signaling, which results in uncontrolled proliferation and metastasis. To address this issue, inhibitors targeting mutant BRAF and MEK, a downstream target of BRAF, have been developed as first-line treatments for melanoma patients with BRAF mutations. Although these drugs are promising in repressing tumor growth, not all patients respond to the treatment, and the benefits are short-term due to the rapid development of resistance [49]. Several studies have demonstrated that metabolic reprogramming is closely associated with resistance to targeted therapies, and targeting metabolic adaptations can delay or overcome the resistance [16].

BRAF-mutant melanoma with resistance to targeted therapies is characterized by increased reliance on OXPHOS, glutamine metabolism, and serine metabolism, which is partially driven by PGC1α [50–53]. Inhibiting the mTORC pathway with AZD8055(or AZD2014) significantly reduced PGC1α levels and resensitized MEKi-resistant melanoma—which exhibited high OXPHOS—to MEKi treatment [50] (Fig. 1). Directly targeting mitochondria with gamitrinib, a small-molecule HSP90 inhibitor, or Phenformin, an inhibitor targeting mitochondrial respiratory complex I, increased apoptosis and cell death in melanoma cells treated with MAPKi [54]. Moreover, OPi, a novel OXPHOS complex I inhibitor, showed antitumor activity in MAPKi-resistant BRAF-mutant melanoma by promoting glycolytic metabolism and suppressing the TCA cycle through the LKB1-AMPK axis [55]. However, growth inhibition and cell death by OPi were not augmented when combined with BRAF or MEK inhibitors in vitro or in vivo. On-steroidal anti-inflammatory drugs (NSAIDs), diclofenac, and lumiracoxib, have been verified to inhibit lactate release and OXPHOS in melanoma cells. Combining NSAIDs with BRAF inhibitors delayed the onset of BRAFi resistance [56]. Nicotinamide adenine dinucleotide (NAD^+^), as a key participant in OXPHOS, was widely elevated &&in BRAFi-resistant melanoma. Consequently, reducing the NAD levels by nicotinamide phosphoribosyltransferase (NAMPT) inhibitors, such as FK866 or GMX1778, has been validated to improve the survival of mice bearing BRAFi-resistant xenografts [57] (Fig. 1). And the increase of OXPHOS in BRAFi-resistant melanoma was mainly dependent on glutamine rather than glucose [53, 58]. Similar to OPi, GLS inhibitor (BPTES or L-DON) monotherapy significantly reduced the growth of BRAFi-resistant melanoma cells, but the antitumor efficacy was not enhanced when combined with vemurafenib, a first-generation BRAFi [53, 58]. Targeting OXPHOS or OXPHOS-dependent carbon sources reduces the survival of BRAFi-resistant melanoma cells, though it does not restore BRAFi sensitivity. However, this metabolic intervention delays the acquisition of resistance in BRAFi-sensitive populations by limiting adaptive metabolic rewiring.

MAPKi-resistant BRAF-mutated melanoma also shows increased FAO and peroxisome biogenesis. Paradoxically, combining the FAO inhibitor etomoxir (ETO) with MAPKi significantly increased tumor growth compared with MAPKi alone [59]. Mechanistically, in ETO-treated melanoma cells, glycolytic flux is increased as a compensatory method of metabolism. However, triple combination therapy with MAPKi, ETO, and dichloroacetate (DCA; a pyruvate dehydrogenase kinase inhibitor that forces glycolytic flux into mitochondria), remarkably delayed tumor relapse in A375P xenografts [59]. Another study demonstrated that targeting FAO with ranolazine (RANO) alone, which blocks acetyl-CoA production from fatty acids in mitochondria, could delay tumor recurrence with acquired BRAFi resistance [60]. Following treatment, tumors also upregulated both glycolysis and OXPHOS to meet energy demands. In addition, (1 *S*, 2 *S*)-2-(2-(*N*-[(3-benzimidazol-2-yl)propyl]-*N*-methylamino)ethyl)-6-fluoro-1,2,3,4-tetrahydro-1-isopropyl-2-naphtyl cyclopropanecarboxylate dihydrochloride (NNC), a small-molecule inhibitor of the PEX3-PEX19 interaction critical for peroxisome biogenesis, restored the sensitivity of MAPKi-resistant melanoma to MAPKi. The combination of NNC with D-threo-PPMP (PPMP), a UDP-glucose ceramide glucosyltransferase(UGCG) inhibitor, further improved the antitumor activity of MAPKi in melanoma models [61].

### Redox modulation and restoration of drug sensitivity

Similar to metastatic melanoma, intracellular redox status in MAPKi-resistant melanoma is also associated with outcomes. Generally, resistant melanoma enhanced the regeneration of reduced GSH by activating the nuclear factor erythroid 2-related factor 2 (NRF2) signaling through the PPP, thereby maintaining redox balance and enabling cell survival [62]. As a transcriptional target of NRF2, thioredoxin reductase 1 (TR1) is important for sustaining the antioxidant capacity of melanoma cells. Although inhibiting TR1 had minimal effect on melanoma growth under nutrient-rich conditions, it truly sensitized melanoma cells to glycolytic metabolism inhibition [63]. Therefore, disrupting the redox balance is an alternative option for melanoma therapy.

Elesclomol, a drug to alter redox balance, had been demonstrated to enhance the cytotoxic effects of paclitaxel, contributing to the reduction of tumor growth, suppression of metastasis, and reversal of therapeutic resistance in preclinical models [64, 65]. However, this phenomenon is observed only in metastatic melanoma patients with normal serum LDH. In advanced melanomas with normal serum LDH, glycolysis and OXPHOS may provide metabolic symbiosis within the same tumor [17]. In contrast, advanced melanoma with high serum LDH primarily relies on glycolysis as its main energy source. Additionally, elesclomol enhanced the cytotoxicity of TH588, a microtubule-modulating agent that induces cell cycle arrest, which relies on endogenous ROS levels [66]. Scavenging ROS with NAC inhibits melanoma metastasis but counteracts the antitumor ability of salidroside (SAL) [67]. Since the tumor-reducing effects of SAL depend on ROS accumulation, leading to mitochondrial dysfunction and ferroptosis. Likewise, cold atmospheric plasma (CAP) also endows melanoma cells with oxidative stress to induce cell death [68, 69]. Targeting Glutathione peroxidase 4 (GPX4), a key enzyme that protects cells against oxidative damage, with deoxyelephantopin (DET) or DET-35 enhances the antitumor activity of vemurafenib against both vemurafenib sensitive and resistant melanoma cells [70] (Fig. 1). Hence, metabolic rewiring plays a nonnegligible role in melanoma response to extrinsic pressure, such as targeted therapy. Understanding the underlying mechanisms helps improve antitumor efficacy and extend the duration of treatment response.

## Metabolism and immunotherapy

### Metabolite-mediated immune evasion

Besides targeted therapy, immunotherapy is a parallel treatment approach for melanoma patients, regardless of BRAF status, by triggering the cytotoxic potential of the powerful immune system to eliminate tumor cells. Unfortunately, the response rates remain relatively low. The tumor microenvironment is considered as a critical factor influencing both the response to immunotherapy and the immune escape of tumor cells. The Warburg effect in melanoma leads to high acidification of the tumor microenvironment, attributed to increased lactic acid production and export [71, 72]. The acidic environment inhibits the infiltration and antitumor function of natural killer (NK) cells, Dendritic cells (DCs), and CD8^+^ T cells [73–77]. Neutralizing tumor acidity with bicarbonate monotherapy significantly reduced tumor growth and largely enhanced antitumor responses to immunotherapy [74]. Moreover, suppression of glycolysis elevated cytotoxic lymphocyte infiltration and improved tumor responsiveness to immunotherapy, in a mouse melanoma model [78]. Qiu et al. developed a tumor-targeted peroxynitrite nanogenerator (APAP-P-NO) that reverses the immunosuppressive tumor microenvironment by selectively impairing metabolic homeostasis in melanoma cells [79].

Metabolites such as acetyl-CoA [80] and inosine [81] also play key roles in regulating immune evasion and immunotherapy response in melanoma. To be more specific, nucleo-cytosolic acetyl-CoA promoted programmed cell death 1 (PD1)-ligand 1 (PD­L1) transcription through P300-dependent histone H3K27 acetylation at the CD274 promoter region. Targeting nucleo-cytosolic acetyl-CoA increased T cell infiltration and enhanced the efficacy of immunotherapy [80]. Additionally, high inosine levels inhibited ubiquitin-like modifier activating enzyme 6 (UBA6) activity, increasing tumor immunogenicity and sensitivity to immune checkpoint inhibition. The combination of inosine with anti-CTLA4 and anti-PD1 antibodies significantly reduced tumor growth in B16-GMCSF mouse models [81].

### Immune cell metabolic reprogramming and antitumor activity

Moreover, the tumor environment imposes metabolic constraints on tumor-infiltrating lymphocytes (TILs), impairing their ability to slow tumor progression [82]. Metabolic reprogramming of TILs is essential for their survival and antitumor function. CD8^+^ TILs exhibited enhanced Peroxisome proliferator-activated receptor alpha (PPAR-α) signaling and fatty acid catabolism in both mouse models and melanoma patients, which is critical for their ability to suppress tumor growth [83]. Additionally, the metabolite phosphoenolpyruvate (PEP) sustained T cell receptor-mediated Ca^2+^-NFAT signaling and effector functions by repressing sarco/ER Ca^2+^-ATPase (SERCA) activity [84]. Increasing PEP production through overexpression of PCK1 enhanced the antitumor ability of TILs. Furthermore, PCK1-overexpressing T cells restricted tumor growth and prolonged the survival of melanoma-bearing mice. Enforced expression of PGC1α, a key regulator of mitochondrial biogenesis and modulates OXPHOS and FAO, in CD8^+^ T cell enhanced antitumor immunity in a mouse melanoma model [85]. Similarly, forkhead box P3 (FOXP3) reprogrammed the metabolism of CD8^+^ T cells, improving their therapeutic efficacy in adoptive T cell therapy.

Dendritic cells (DCs) also play a crucial role in initiating and regulating antitumor immune responses. However, tumors can hijack DCs through the release of immunosuppressive factors and immunomodulatory molecules, thereby impairing their antitumor functions. In addition, tumor-associated glycans can alter the metabolism of DCs, further suppressing their immunostimulatory capacity. Blocking MCT1 with BAY8002 can prevent the metabolism of DCs from skewing by tumor-derived glycans and restore their capacity to support effective antitumor T cell responses [77]. In tumors resistant to anti-PD1 therapy, DCs exhibit elevated mitochondrial respiration and FAO, accompanied by a reduced ability to stimulate T cells. Targeting MerTK has been shown to modulate DC metabolism and function, thereby enhancing anti-PD1 therapy [86].

## Conclusion and perspectives

Melanoma metabolism exhibits remarkable plasticity, supporting tumor growth and proliferation in different stages and environments. This metabolic flexibility also enables melanoma cells to adapt and survive under various treatment stresses, contributing to therapy resistance. Targeting the metabolism of melanoma can inhibit melanoma growth and delay or entirely overcome treatment resistance (Table 1), and combining metabolic interventions with other treatments enhances antitumor efficacy (Table 2). Numerous drugs targeting metabolic pathways are currently under development or in clinical trials for cancer treatment [6]. However, targeting metabolic pathways may lead to unintended toxicity in normal cells that share similar metabolic dependencies with tumor cells. For example, 2DG has been reported to induce adverse effects such as fatigue, sweating, dizziness, and hypoglycemia [87]. ETO can impair the function of immune cells, including macrophages and T cells [88]. To minimize off-target toxicity, optimizing dosage, strategies such as dose optimization, targeted delivery systems, and the design of tumor-selective agents should be considered.

**Table 1:** Compounds targeting melanoma metabolism.

Drug	Target	Effects on melanoma	Reference
**2DG**	Glucose metabolism	Inhibit growth	[20, 23]
**MTyr-OANPs**	glycolysis	Inhibit growth	[12]
**SR-18292**	PGC1α	Inhibit tumorigenicity, growth, and invasion	[31]
**XCT790**	PGC1α	Inhibit tumorigenicity, growth, and invasion	[31]
**NAC**	ROS	promoted distant metastasis	[32]
**Methotrexate**	Folate pathway	Inhibit distant metastasis	[32]
**Etomoxir**	FAO	Inhibit cell migration	[39]
**RANO**	FAO	Delay tumor recurrence	[60]
**2-AAPA**	glutathione reductase	Inhibit distant metastasis	[33]
**AZD8055 or AZD2014**	mTORC/OXPHOS	Resensitized MEKi-resistant melanoma	[50]
**FK866 or GMX1778**	nicotinamide phosphoribosyltransferase	Inhibit growth	[57]
**Gamitrinib**	Hsp90	Inhibited acquired resistance	[54]
**Phenformin**	Complex I	Inhibit growth, induce cell death	[54]
**OPi**	Complex I	Inhibit growth, induce cell death	[55]
**BPTES**	GLS1	Inhibit growth, inhibit cell migration	[13, 58]
**L-DON**	GLS	Inhibit growth, inhibit cell migration	[58]
**CB-839**	GLS	Inhibit growth	[7]

**Table 2:** Combination therapy of metabolic inhibitors.

Metabolic inhibitor	Treatment	Model	Reference
**2DG**	cellulose nanocrystal & doxorubicin (DOX)	B16F10 tumor-bearing mice	[23]
**2DG**	Sorafenib	NRAS^Q61^ mutates melanoma cells	[24]
**2DG**	Coronatine	Metastasis melanoma cells	[21]
**2DG**	cisplatin or staurosporine	Metastasis melanoma cells	[11]
**2DG**	Pyruvate	Metastasis melanoma cells	[11]
**2DG**	TRAIL	Melanoma cells	[22]
**Gamitrinib**	MAPKi	1205Lu, WM9 cells	[54]
**NNC**	MAPKi	Melanoma cells, mouse model	[61]
**NNC** **+** **PPMP**	MAPKi	Melanoma cells, mouse model	[61]
**Etomoxir** **+** **DCA**	MAPKi	A375P xenografts	[59]
**Elesclomol**	paclitaxel	Melanoma cells	[65]
**FK866 or GMX1778**	dabrafenib	Xenograft models	[57]
**BPTES**	PLX4720	A375	[53]
**MTyr-OANPs**	photothermal therapy	B16F10 tumor-bearing mice	[12]

The metabolic flexibility of melanoma cells enabled them to adapt to therapeutic pressure by rerouting their metabolism through alternative pathways, which limits the effectiveness of therapies targeting a single metabolic pathway. For example, the loss of viability caused by PGC1α suppression in melanomas could be rescued by the induction of glycolysis through hypoxia-inducible factor-1a (HIF1a) [89]. Similarly, MAPKi-treated melanoma cells increase glycolysis to overcome the metabolic stress induced by FAO inhibition [59]. And studies have shown that targeting multiple metabolic pathways could enhance antitumor efficacy. For instance, dual suppression of PGC1α and HIF1a caused energetic deficits and loss of viability that are partially compensated by glutamine utilization. Notably, triple suppression of PGC1α, HIF1a, and glutamine utilization resulted in complete blockage of tumor growth. Concomitant inhibition of FAO and glycolysis enhances the efficacy of MAPKi therapy. Targeting multiple metabolic pathways represents a promising strategy to improve antitumor efficacy by disrupting the metabolic flexibility of melanoma cells and overcoming therapy resistance.

The environment is a critical factor for influencing the metabolic state of melanoma cells and modulating the antitumor efficacy of metabolic inhibitions. Suppressing glycolysis induced by PFKFB2 mutation significantly reduced tumor growth in nude mice, while not impairing melanoma cell proliferation under normal in vitro culture conditions [18]. Studies have shown that mitochondria can be shuttled intercellularly and influence cell metabolism [90, 91]. Melanoma cells transferred mitochondria to T cells, inducing metabolic abnormalities and senescence in T cells [91]. The influence of the tumor microenvironment on metabolic adaptations cannot be ignored, as it plays a critical role in shaping cellular responses and therapeutic outcomes.

The metabolism of melanoma significantly influences treatment options. For example, elesclomol enhanced the cytotoxic effects of paclitaxel specifically in melanoma cells with high OXPHOS [65]. Serum LDH levels can serve as an indicator of melanoma metabolism and predict sensitivity to elesclomol. Similarly, metabolomic profiling of peripheral blood mononuclear cells (PBMCs) has been proposed as a potential biomarker for predicting the response to immune checkpoint inhibitor therapy. Melanoma patients who respond to treatment typically exhibit PBMC with higher reserve respiratory capacity and glycolytic activity [92]. In addition, metabolic parameters derived from 2-fluorodeoxyglucose positron emission tomography/computed tomography (2-[18 F]FDG PET/CT) are highly correlated with the overall survival of patients undergoing immunotherapy [93–96]. Metabolic characteristics hold promise as biomarkers for selecting treatment strategies and monitoring, offering a personalized approach to melanoma therapy.
